# Palliative External Beam Radiation Therapy for Dysphagia in a 101-Year-Old Man With Esophageal Adenocarcinoma

**DOI:** 10.7759/cureus.100837

**Published:** 2026-01-05

**Authors:** Pericles J Ioannides, Jester M Odrunia, Gina N Perez, Morgan Butow, Georg A Weidlich

**Affiliations:** 1 Radiation Oncology, Adventist Health, Sonora, USA; 2 Medical Physics, Zap Surgical Systems, San Carlos, USA

**Keywords:** esophageal adenocarcinoma (eac), esophageal cancer, gastroenterology palliative, geriatric patient population, nonsurgical treatment, palliative radiation therapy, supportive and palliative care, swallowing impairment, targeted radiation therapy, unintentional weight loss

## Abstract

Esophageal adenocarcinoma in centenarians is rare, and treatment options in this age group are limited. Dysphagia is often the predominant symptom that affects quality of life (QoL), and options for treatment and palliation are limited when patients are not candidates for chemoradiation and endoscopic interventions fail. We present the case of a 101-year-old male patient with symptomatic distal gastroesophageal junction (GEJ) adenocarcinoma who presented with progressive dysphagia, aspiration, and weight loss. Esophagogastroduodenoscopy (EGD) was performed to confirm the diagnosis and to place a metal stent for obstruction. He was deemed medically unsuitable for definitive chemoradiation or surgery, with goals of care to avoid an enteric feeding tube for nutrition and prolong life while maintaining his QoL. The metal stent had migrated, providing limited improvement and progression in dysphagia, and physicians in a multidisciplinary review recommended that he proceed with either hospice or palliative radiation therapy. The patient underwent palliative external beam radiation therapy (EBRT) to a total dose of 37.5 Gy in 15 fractions, targeting the obstructive esophageal mass with a margin using volumetric-modulated arc therapy (VMAT) technique. The treatment was well tolerated by the patient with no significant acute toxicity. After treatment, the patient reported clinically meaningful improvement in functional status, with improved swallowing, advancing from liquids to a soft diet, weight gain, and improved QoL. Moderate-dose palliative radiation can be a safe and effective treatment in a centenarian with obstructive GEJ adenocarcinoma, particularly when endoscopic interventions are not successful or durable. This report shows the importance of shared decision-making in geriatric oncology, where performance status and goals of care guide the selection of treatment options, and supports the use of palliative EBRT in the centarian population as a successful modality to improve swallowing and QoL in carefully selected patients with dysphagia secondary to malignancy.

## Introduction

Esophageal cancer is the ninth most common cancer diagnosis and the sixth most common cause of cancer death worldwide, with adenocarcinoma rising in incidence as a global health burden [1-2]. 

Dysphagia is known to impair oral intake of adequate nutrition, leading to decreased functional status and quality of life [3]. In patients who are not candidates for curative-intent treatment, palliative treatments are offered to improve organ function and quality of life [4]. Immediate intervention to alleviate obstruction with upper endoscopy and self-expandable metallic stents (SEMS) can be used to relieve obstruction; however, stent migration and tumor overgrowth limit the durability of symptom relief and control [5]. External beam radiation therapy (EBRT) for palliation of dysphagia is an established treatment option and may be used to improve long-term durability compared to stenting alone [6]. 

The management of cancer in older and frail patients includes establishing goals of care for treatment intent, symptom control, social and spiritual needs, advance directives, and end-of-life care [7]. Evidence in guiding decision-making for these patients is sparse, and reports of palliative EBRT in centenarians are rarely reported in the literature. 

We report on the benefit of moderate-dose palliative radiation as a safe and effective treatment in a centenarian with obstructive gastroesophageal junction (GEJ) adenocarcinoma, particularly when endoscopic interventions are not successful or durable. It shows the importance of shared decision-making in geriatric oncology, where performance status and goals of care guide the selection of treatment options. This case supports the use of palliative EBRT in the centarian population as a successful modality to improve swallowing and quality of life (QoL) in carefully selected patients with dysphagia secondary to malignancy.

## Case presentation

A 101-year-old man, with stage III cT3N0M0 esophageal adenocarcinoma with a history of gastroesophageal reflux disease (GERD), hypertension, hyperlipidemia, atrial fibrillation, and age-related frailty (Eastern Cooperative Oncology Group (ECOG) 3), presented with three months of worsening dysphagia to solids then liquids, poor nutrition intake, aspiration pneumonia, and unintentional weight loss. 

On May 15, he underwent a computed tomography (CT) scan of the chest, abdomen, and pelvis, which revealed a distal esophageal mass extending into the GEJ. An esophagogastroduodenoscopy (EGD) performed the same day demonstrated a circumferential, friable tumor located at 38 cm from the incisors, and biopsy confirmed moderately differentiated esophageal adenocarcinoma, stage III cT3N0M0.

On May 20, a self-expanding metal stent was placed for relief of symptomatic dysphagia; however, the stent failed by migrating shortly after placement, resulting in no durable improvement. The patient then had three EGD-guided ablations on May 22, May 27, and May 30, with partial symptom relief. Medical oncology concluded that due to advanced age, frailty, and comorbidity, he was not a candidate for concurrent chemoradiation. After goals of care discussion for hospice versus palliative measures, he wished to proceed with palliative care with goals of DNR and goals not to have an enteric feeding tube; his oncology providers recommended palliative radiation therapy (EBRT). 

**Figure 1:**
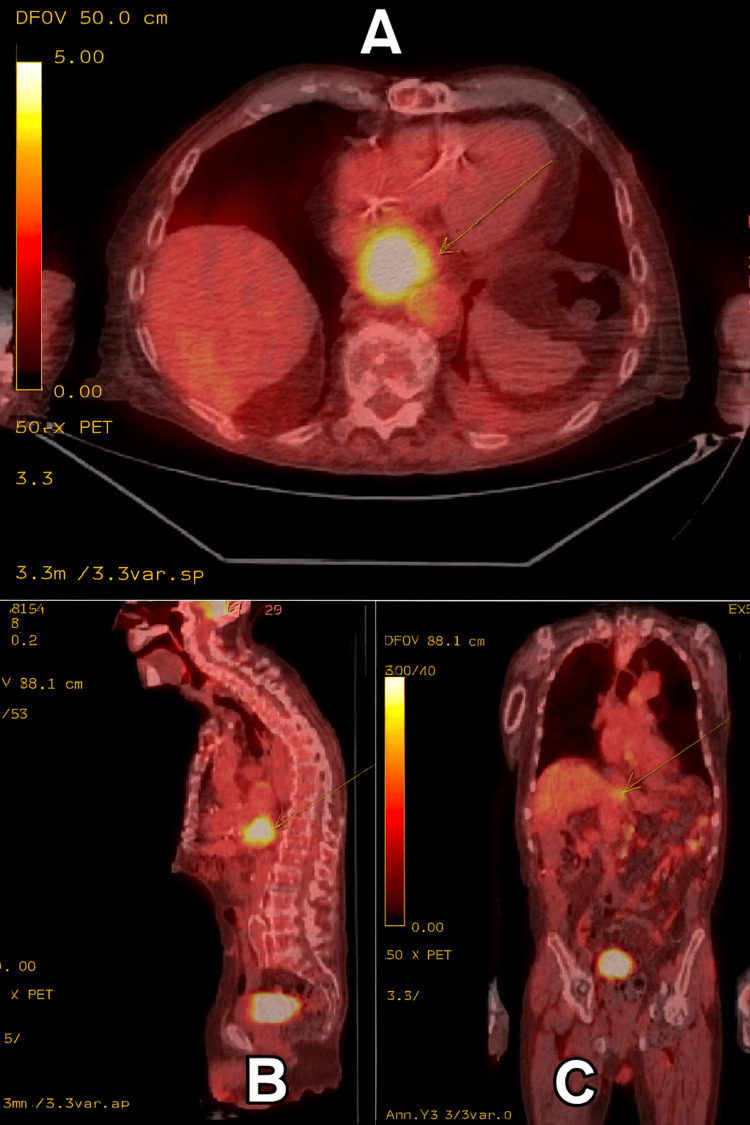
Axial (A), sagittal (B), and coronal (c) view of 18FDG-PET scan of the patient with arrows showing uptake limited to the distal esophagus 18F-FDG: fluorodeoxyglucose

On June 10, he presented for radiation oncology consultation, where, after shared decision-making with the patient and family, palliative EBRT was recommended for improving dysphagia, nutrition status, enteric feeding tube free survival, and QoL. The patient reported progressive dysphagia with a Mellow-Pinkas Dysphagia Score of 3 (liquids only) at presentation. On June 30, PET-CT showed uptake confined to the distal esophagus with no evidence of regional or distant metastasis (Figure 1).

**Figure 2:**
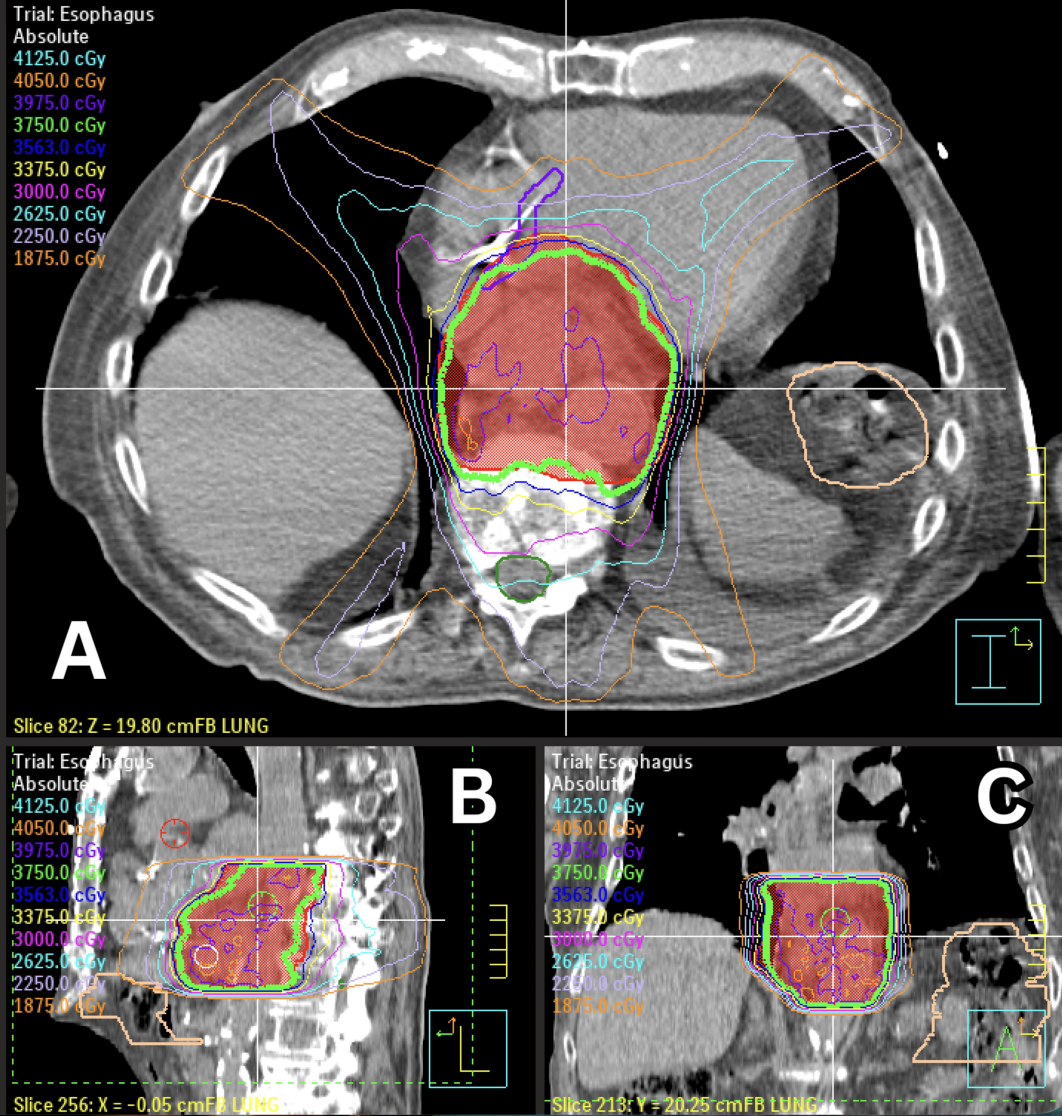
Isodose distribution of axial (A), sagittal (B), and coronal (C) orientation. The prescription isodose line (green) represents the dose distribution of 3750 cGy.

**Figure 3:**
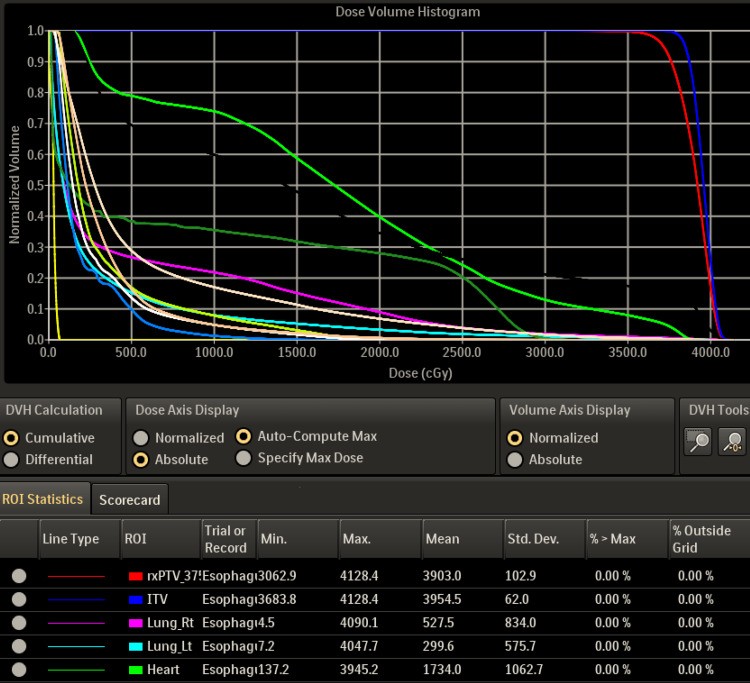
Dose volume histogram (DVH) Planning target volume (red), internal target volume (ITV), and critical structure dose for the heart (green), right lung (pink), and left lung (light blue).

A palliative regimen of 37.5 Gy in 15 fractions targeting the obstructive esophageal neoplasm with a margin using volumetric modulated arc therapy (VMAT) technique was completed by the patient from July 1 to July 25. Figure 2 illustrates the dosimetric treatment plan with color-coded isodose lines. The dose volume histogram (DVH) is shown in Figure 3, and planning dose objectives in Table 1. 

**Table 1:** Planning objectives and organs at risk (OAR) The radiation treatment planning constraints, the prescribed tumor coverage dose, and the surrounding organ-at-risk values are used to limit radiation exposure to normal tissues. DVH: dose volume histogram; PTV: planning target volume

Structure	Objective	Dose Value (cGy)	Criteria
PTV 3750	Minimum DVH (%)	3750	95%
Spinal Canal	Maximum Dose	4200	Point Dose
Spinal Canal	Maximum DVH (ccm)	3900	5.0 ccm
Total Lung	Maximum DVH (ccm)	1650	1500 ccm
Total Lung	Maximum DVH (%)	1800	37.0 %
Heart	Maximum DVH (ccm)	4890	0.035 ccm
Heart	Maximum DVH (ccm)	4200	15.0 ccm
Liver	Maximum DVH (ccm)	3000	700.0 ccm
Kidneys	Maximum DVH (ccm)	2400	200 ccm

**Figure 4:**
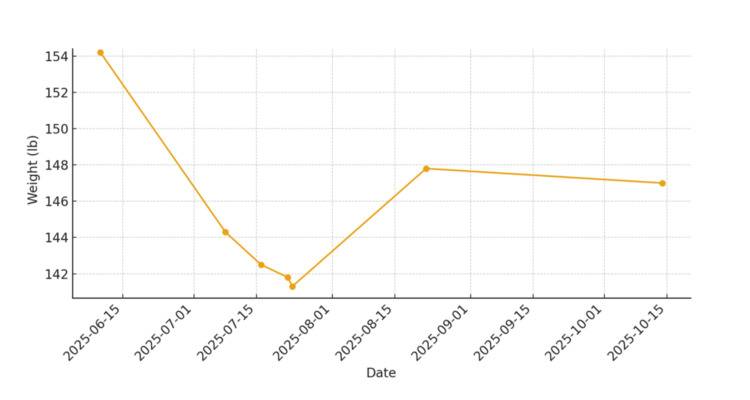
The patient's weight at consultation, before, during, and after treatment

The patient's baseline weight was evaluated at consultation, weekly on treatment, and then post treatment. While on treatment, he experienced weekly improvement in swallowing and reduced aspiration and cough, with no acute grade ≥2 toxicities. His pre-treatment weight showed a transient decline during treatment, followed by improvement towards the end and at post-treatment follow-up, as seen in Figure 4.

**Figure 5:**
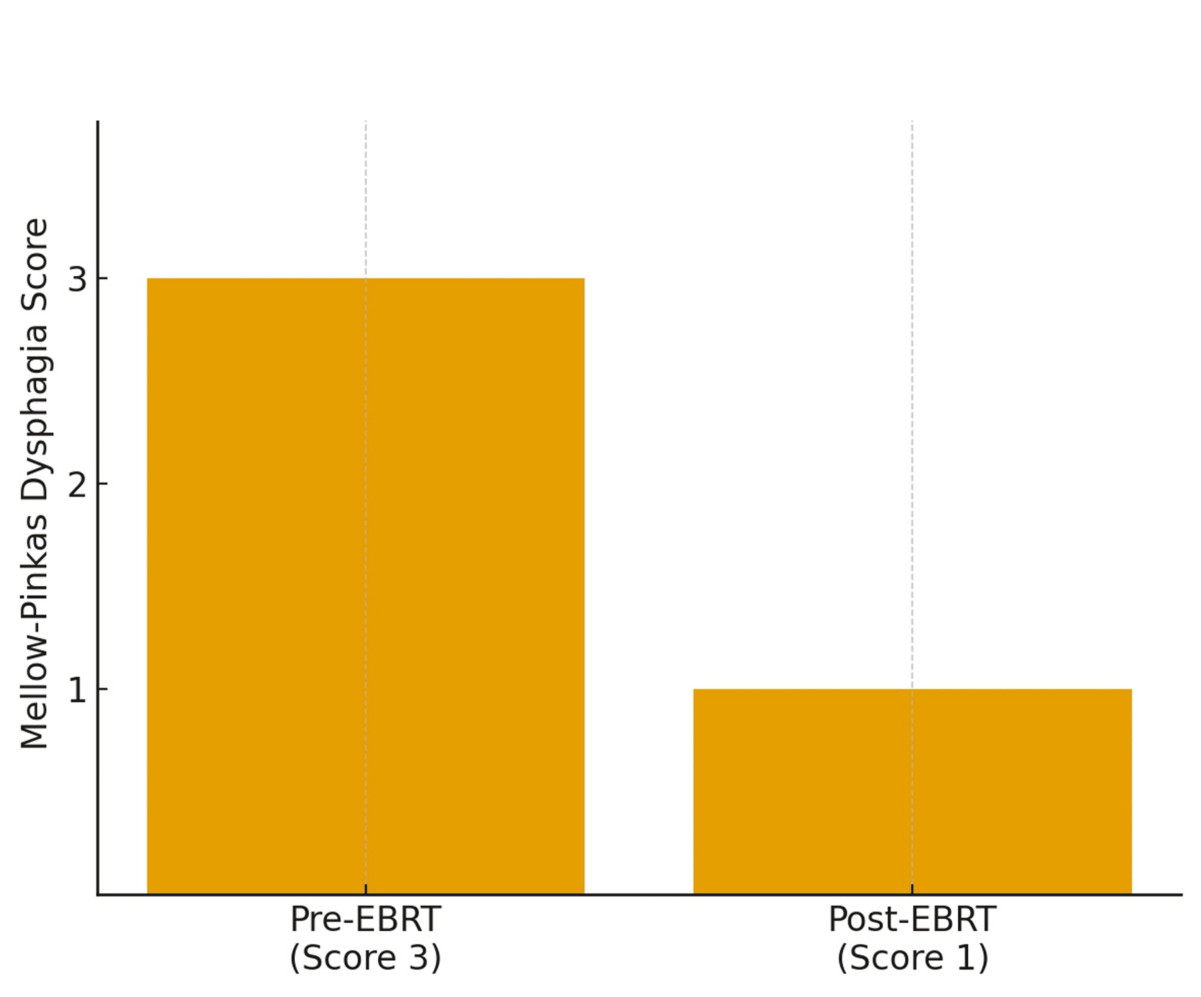
Mellow-Pinkas dysphagia score before and after palliative radiotherapy EBRT: external beam radiation therapy

Following radiation therapy, his Mellow-Pinkas dysphagia score (Table 2) improved to 1 (able to tolerate some solids) as seen in Figure 5.

**Table 2:** Mellow–Pinkas scoring system for dysphagia

Grade	Criteria
0	Able to eat a normal diet/no dysphagia
1	Able to swallow some solid foods
2	Able to swallow only semi-solid foods
3	Able to swallow liquids only
4	Unable to swallow anything/total dysphagia

His goals of care were met with improved swallowing, decreased aspiration, and maintenance of his ability to swallow without an enteric feeding tube. He was offered routine follow-up in the radiation oncology department with supportive care and palliative measures monitored by his primary care physicians.

## Discussion

The treatment of esophageal adenocarcinoma in the geriatric population, and especially in the centenarian population, presents significant challenges in care. Curative intent therapies such as definitive chemoradiation surgery are often not suitable due to functional status and frailty [8]. EBRT with palliative intent remains a viable treatment with the intent to relieve pain, obstruction, bleeding, and improve QoL [9].

In this case, palliative EBRT was able to provide relief of dysphagia and aligned with the patient's goals of enteric feeding tube-free survival rather than solely pursuing oncologic control of disease or enrolling in hospice prior to relief of his symptoms. Intervention with upper endoscopy, SEMS, and the use of ablative procedures are commonly used for rapid palliation. However, these options are limited by early stent migration, tumor overgrowth, discomfort, and reduced durability [10,11]. Following stent migration and only partial improvement with ablative treatments, alternative modalities such as EBRT were needed to provide the patient with sustainable palliation.

EBRT is a well-established palliative option, with studies reporting symptomatic dysphagia improvement in approximately 60-80% of patients, consistent with our patient's response to treatment [12,13]. The regimen used in this case, 37.5 Gy in 15 fractions, was chosen over other standard regimens of 30 Gy in 10 fractions or 20 Gy in five fractions to fall within a higher biologically effective dose (BED) range associated with potentially more durable symptom control while remaining tolerable for a frail, extremely elderly patient. Multiple analyses of treatment of dysphagia have shown that higher BED regimens (>30-35 EQD2) have more sustained dysphagia relief in terms of durability and magnitude, likely secondary to improved tumor cytoreduction and delayed regrowth compared to lower BED regimens [13-17]. Additionally, this regimen was chosen given the patient's life expectancy of more than three months with a single site of disease on staging imaging, consistent with broad guidance that favors longer-course EBRT when life expectancy exceeds three months. VMAT-based delivery was used to further improve the therapeutic ratio by enhancing conformality and reducing dose to adjacent organs. 

Given the subjectivity of dysphagia symptoms, objective dysphagia scoring systems such as the Mellow-Pinkas scale were used to quantify the impact of palliative radiation in a standardized way to be compared across other clinical studies and reports [18]. Incorporating such assessment tools helps contextualize symptom improvement after both endoscopic and radiation-based approaches. Consistent with this, the patient's dysphagia score improved from 3 at baseline to 1 after radiotherapy.

The patient's treatment highlights the use of established geriatric oncology guidelines, which recommend that oncologists base treatment decisions on functional status, life expectancy, and goals of care rather than chronological age alone [19,20]. Our patient priorities of symptom relief and enteric feeding tube-free survival were met using goal-directed EBRT as a non-invasive, outpatient-based, tailored approach designed for symptom relief and to minimize toxicity. 

This case report contributes to the literature supporting the use of palliative EBRT for geriatric patients ineligible for definitive therapy. The patient experienced improvement in swallowing without significant toxicity, supporting the geriatric oncology guidelines that chronological age alone should not preclude palliative EBRT. For patients who are ineligible for definitive chemoradiation or surgery and have not had a durable response to endoscopic intervention, palliative EBRT remains a viable and durable option to improve dysphagia and QoL. 

## Conclusions

This case demonstrates that palliative EBRT can provide meaningful and durable dysphagia relief even in patients of advanced age, including centenarians. Despite the limitations of endoscopic stent migration and incomplete response to ablative procedures, palliative EBRT delivered with VMAT resulted in clinically significant improvement in swallowing, without appreciable toxicity.

The favorable response seen in the patient after treatment highlights the importance of goal-directed care in geriatric oncology, rather than chronological age alone. This case shows the benefit of EBRT in carefully selected patients and adds to the limited evidence for the use of non-invasive EBRT in the centenarian population as an effective method for palliation. Clinical providers should remain open to the option of offering EBRT in patients with a similar presentation, particularly when other interventions have failed and when the patient desires durable improvement in dysphagia through shared decision-making and goals of care. 
